# Can Genetic Predisposition Play a Role in Anti‐Glutamic Acid Decarboxylase (GAD) Autoimmunity? A Rare Presentation in Three Siblings

**DOI:** 10.1155/crii/6581645

**Published:** 2026-04-25

**Authors:** Ammar Alobaidy, Mulham Alsulaimi, Ameer Alajmi

**Affiliations:** ^1^ Department of Medicine-Neurology Unit, Sultan Qaboos University Hospital, Muscat, Oman, squh.edu.om; ^2^ College of Medicine and Health Sciences, Sultan Qaboos University, Muscat, Oman, squ.edu.om

## Abstract

Anti‐glutamic acid decarboxylase (anti‐GAD) autoimmunity possess a poorly understood diversity in the development of neurological manifestations with or without diabetes mellitus (DM) association. A possible genetic contribution might further expand the complexity of anti‐GAD pathogenesis and demand further exploration and research. We report three siblings in the same family (two sisters and one brother) who were tested positive for anti‐GAD antibodies (anti‐GAD‐Abs) and presented with different clinical disorders, associated with DM in one of them. Although scarce data are available in the literature concerning the influence of possible genetic predisposition on the development of anti‐GAD autoimmunity, yet there is cumulative evidence to support this association which is further elucidated in this report. Whether to recommend family screening tests for patients with positive anti‐GAD autoimmunity is still undetermined and further studies are required to solve the unanswered queries pertinent to this potentially treatable autoimmune disorder.

## 1. Introduction

Anti‐glutamic acid decarboxylase (anti‐GAD) autoimmunity is a peculiar autoimmune disorder that can affect the pancreas and nervous system, resulting in a spectrum of various neurological presentations that may or may not be associated with development of diabetes mellitus (DM) [1]. Different pathomechanisms had been hypothesized to explain the development of anti‐GAD autoimmunity, yet the genetic contribution as an etiological factor is not well studied in the literature [2].

Recently, we published a study that explored the spectrum of neurological manifestations, existence of DM, and 5‐year mortality and cancer association outcomes in a cohort of Omani patients with positive anti‐GAD autoimmunity [1]. The study retrospectively enrolled 444 GAD‐antibodies (GAD‐Abs) positive Omani patients and evaluated the correlations between GAD‐Ab titers and the presence of neurological manifestations and/or DM, with a 5‐year follow‐up to determine the mortality and cancer association outcomes [1]. While exploring the electronic medical records of patients with positive GAD‐Abs testing during our data collection, we found that three members of the same family (two sisters and one brother) had positive GAD‐Abs with different systemic and neurological manifestations as described below, which highlights the need to explore for a possible genetic predisposition in anti‐GAD autoimmunity. In all three cases, serum anti‐GAD‐Abs were determined using two complementary assays from Euroimmun Medizinische Labordiagnostika AG (Lübeck, Germany). First, a quantitative enzyme‐linked immunosorbent assay (ELISA) was employed; according to the manufacturer’s protocol, a GAD‐Ab titer of 10 U/mL was defined as positive [1]. To assess the clinical significance, end‐point titrations were recorded, revealing high‐titer levels of >2000 U/mL in Case 1 and Case 2, and an elevated level of 240 U/mL in Case 3.

Second, a qualitative Western blot (immunoblot) immunoassay was performed to confirm antibody specificity. This involved the use of cellulose membrane strips precoated with purified GAD antigens. After incubation with patient serum, the binding of anti‐GAD‐Abs was visualized through an enzymatic color reaction, providing definitive verification of the ELISA findings [1].

## 2. Case Presentation

**Figure 1:**
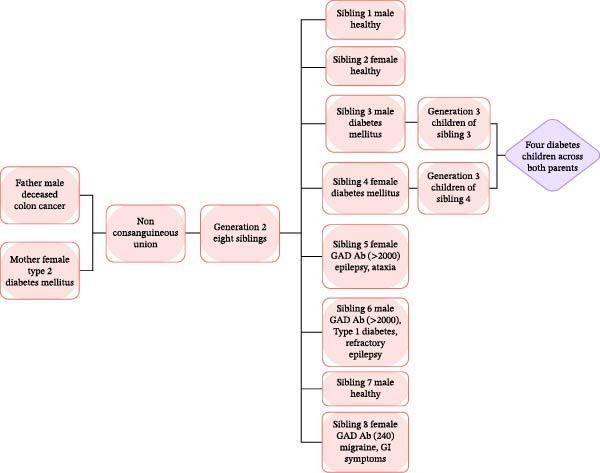
Family medical tree illustrating the familial clustering of glutamic acid decarboxylase (GAD) antibody autoimmunity and diabetes mellitus. The chart highlights eight siblings in Generation II, featuring three key cases (Siblings 5, 6, and 8) with elevated GAD‐Ab titers associated with varying neurological and endocrine manifestations, including refractory epilepsy, ataxia, and type 1 diabetes. The diagram further demonstrates a strong multi‐generational history of diabetes, encompassing the mother (Generation I), two additional siblings (Generation II), and four grandchildren (Generation III). GAD Ab, glutamic acid decarboxylase antibodies; GI, gastrointestinal.

The family structure and medical history are represented in the family medical tree (Figure 1). The family consists of non‐consanguineous parents and eight offspring. The maternal history is significant for type 2 DM, and the paternal history includes colon cancer. Among the eight siblings, three (ranks 5, 6, and 8) tested positive for GAD‐Abs with varying clinical presentations. Notably, the familial clustering of metabolic dysfunction extends beyond the indexed cases: two other siblings and four of their children also have a clinical history of DM, suggesting a strong hereditary predisposition to autoimmune and metabolic disorders. The first case with positive GAD autoimmunity in the family is a female rank fifth among the siblings, in late 40s, known to have nocturnal epilepsy for 10 years on antiseizure medications, presented with gradual onset and slowly progressive gait ataxia and short‐term memory difficulties over the last few months before her presentation to the hospital. Her clinical and neurological examination was remarkable for cerebellar gait ataxia and impaired delayed recall in Montreal Cognitive Assessment. Investigations were remarkable for positive serum GAD‐Abs by serum ELISA (>2000 U/mL) and immunoblot tests. Other autoimmune screening tests revealed positive serum anti‐thyroid peroxidase (TPO) antibodies with normal thyroid function tests (T4 and TSH). Brain MRI showed nonspecific white matter changes. Other investigations for diabetes and malignancy were unremarkable, including pan CT scans (chest, abdomen, and pelvis) and limbic/paraneoplastic autoantibodies in the serum and cerebrospinal fluid (Table 1).

**Table 1:** Comparison between the cases.

Siblings	Background	Presentation	Screening for other autoimmune disorders and malignancy	Brain MRI findings
Case 1: female in late 40s	Epilepsy; no other comorbidity	Cerebellar ataxia	Positive anti‐TPO, normal T4, TSH Unremarkable malignancy screening tests	Nonspecific white matter changes
Case 2: male in mid 40s	Type 1 diabetes; no other comorbidity	Epilepsy refractory to medications	Anti‐gastric parietal cell and anti‐TPO. Normal B12, T4 and TSH Unremarkable malignancy screening tests	White matter changes suggestive of small vessels disease
Case 3: female in late 30s	Chronic migraine; no other comorbidity	Abdominal pain and diarrhea	Positive ANA Unremarkable malignancy screening tests	Nonspecific white matter changes

The second case is a male rank sixth among the siblings, in mid 40s, known to have DM type 1 started when he was in early 30s, presented with a new onset focal epilepsy with secondary generalization, refractory to antiseizure medications. General and systemic examination was unremarkable after recovery from the seizures. Investigations showed positive GAD‐Abs by serum ELISA (>2000 U/mL) and immunoblot tests. Other autoimmune screening tests revealed positive serum anti‐gastric parietal cell and anti‐TPO antibodies. B12 level and thyroid function tests were normal. Brain MRI showed white matter changes suggestive of small vessel disease. Other malignancy screening investigations were unremarkable (Table 1).

The third patient is a female rank eighth among the siblings, in late 30s, diagnosed with chronic migraine headache for 10 years, presented with a 1‐year history of abdominal pain and diarrhea. General and systemic examination was unremarkable. Investigations showed elevated serum GAD‐Abs titer by ELISA only (240 U/mL), unremarkable diabetes and other autoimmune screening tests, apart from positive ANA (titer 1/640) with negative antibodies against nuclear‐DNA and extractable nuclear antigens. Brain MRI showed nonspecific white matter changes (Table 1).

The first case was started on immunotherapy in the form of oral steroids, intravenous immunoglobulin (IV IgG) and rituximab, with good response in gait ataxia and memory challenges. In addition, a better control of seizures was also observed and currently she is regularly taking rituximab every 6 months. Follow‐up GAD‐Abs titer was not done after starting the immune therapy (Table 2).

**Table 2:** Outcome and follow‐up.

Cases	Initial anti‐GAD titer	Treatment	Follow‐up anti‐GAD titer	Outcome
Case 1	>2000 U/mL	Steroids, IV IgG, rituximab	Not available	Improvement in symptoms with better epilepsy control
Case 2	>2000 U/mL	IV IgG and rituximab	>2000 U/mL	Improvement in seizures frequency, but started to have cerebellar ataxia 2 years after the diagnosis and use of immunotherapy
Case 3	240 U/mL	Not started	Not available	Unremarkable gastrointestinal tract investigations. Started on migraine prophylaxis medication

The second case was started on immunotherapy (immunoglobulin and rituximab) and his seizures frequency improved from several seizures per week to 1–2 seizures per month. Steroid treatment was not used in view of his diabetes. However, he started to have cerebellar gait ataxia with persistent high titer GAD‐Abs despite receiving rituximab for 2 years. No further follow‐up information could be retrieved from the patient’s electronic records as he decided to continue the management at a different healthcare facility and he did not show up for the subsequent scheduled appointments (Table 2).

The third case underwent the following investigations: upper and lower gastrointestinal tract endoscopy, abdominal ultrasound, and celiac disease screening, with unremarkable results. She was started on migraine prophylaxis, and no immune therapy started for her (Table 2).

## 3. Discussion

Scarce data can be found in the literature concerning the influence of possible genetic predisposition on the development of anti‐GAD autoimmunity [3]. Similar to our cases, this rare familial clustering of GAD‐Abs positive patients was also reported in the literature in a father and daughter with stiff‐person syndrome, two sister siblings with cerebellar ataxia and other familial case reports of stiff‐person syndrome [4–6]. Polyendocrine autoimmunity in association with GAD‐Abs was also reported in the literature [7], similar to the associated positive thyroid autoimmunity in two of our patients. The clinical profile of this family, particularly Case 2 (type 1 DM, anti‐TPO, and anti‐gastric parietal cell antibodies) and Case 1 (anti‐TPO antibodies), raises the question of autoimmune polyendocrine syndrome Type 2 (APS 2) or a related polyautoimmunity variant. The presence of high‐titer GAD‐Abs in this context may act as a common denominator between endocrine and neurological manifestations, reinforcing the need for broader autoimmune screening in families with a strong history of type 1 DM. A more recent, interview‐based study explored the autoimmune diseases displayed by the relatives of patients having GAD‐Abs associated neurological syndromes and found that 44 out of 65 patients (67.7%) reported family history of autoimmunity, including first‐degree relatives in 36/65 (55.4%), and the sibling recurrence risk was 5.5, suggestive of an underlying potential genetic predisposition [3]. Exploratory genetic studies detected some rare human leukocyte antigen (HLA Class II) haplotypes, as well as potential candidate variants in genes linked to autoimmunity or representing immunological check‐points that might have an implication in developing DM and/or neurological manifestations in anti‐GAD autoimmunity [8, 9]. However, these studies were too small to draw general conclusions [8]. A more recent large cohort of 1214 individuals, including 167 patients with sporadic GAD‐Abs autoimmune neurological syndromes, was conducted in Germany by using a genome‐wide association study and analysis of the HLA region [10]. This study identified 16 genome‐wide significant loci for the susceptibility to neurological manifestation of anti‐GAD autoimmunity. The top variant localized to an intergenic segment in the middle of the HLA Class I region and over 40% of the variants have known regulatory functions on the expression of 48 genes in disease relevant cells and tissues, mainly CD4^+^ T cells and the cerebral cortex [10].

The study suggested that variations at multiple genes involved in innate and adaptive immunity, as well as contributing to neural structure and function, modulate the susceptibility to anti‐GAD autoimmune neurological syndromes [10].

As a limitation in our case report, genetic testing was not performed. However, the lack of clearly defined genetic loci for possible genetic contribution in anti‐GAD autoimmunity might influence the decision to send for genetic testing; in addition, previous case reports also did not include genetic testing, probably for the same reason [5–7].

Furthermore, the size of this family—consisting of eight siblings with three confirmed GAD‐Abs positive cases—presents an ideal candidate for linkage analysis or whole exome sequencing (WES). Identifying such multiplex families is a vital first step in detecting rare HLA Class II haplotypes or candidate variants in immunological checkpoints. Although genetic testing was not performed in this instance, the high sibling recurrence risk (5.5) reported in similar cohorts suggests that such familial clusters are essential for defining the genetic determinants that modulate the susceptibility to anti‐GAD neurological syndromes.

As a conclusion, GAD‐Abs associated disorders represent a spectrum of manifestations, possibly due to autoimmune recognition of different GAD epitopes with a diverse genetic background. This case report and brief literature review illustrate the cumulative evidence to suggest a genetic predisposition in anti‐GAD autoimmunity with direct implications for development of neural dysfunction with or without DM association. Whether to recommend family screening for symptomatic patients with positive GAD‐Abs testing, is still a question that needs to be addressed through larger prospective studies.

## Author Contributions

Ammar Alobaidy contributed to the conceptualization, drafting, and revising the manuscript and was involved in clinical care of the patients. Mulham Alsulaimi and Ameer Alajmi contributed to the data collection and analysis and revising the manuscript.

## Funding

No funding was received for this manuscript.

## Disclosure

All authors approved the final manuscript as submitted and agree to be accountable for all aspects of the work.

## Consent

Written informed consent was obtained from the patients for publication of this case report.

## Conflicts of Interest

The authors declare no conflicts of interest.

## Data Availability

Data sharing is not applicable to this article as no datasets were generated or analysed during the current study.
